# The Supernatant of Tonsil-Derived Mesenchymal Stem Cell Has Antiallergic Effects in Allergic Rhinitis Mouse Model

**DOI:** 10.1155/2020/6982438

**Published:** 2020-04-07

**Authors:** In-Su Park, Ji Hye Kim, Jun-Sang Bae, Dong-Kyu Kim, Ji-Hun Mo

**Affiliations:** ^1^Ajou University Medical Center, Suwon, Republic of Korea; ^2^Department of Otorhinolaryngology, Dankook University College of Medicine, Cheonan, Republic of Korea; ^3^Beckman Laser Institute Korea, Dankook University College of Medicine, Cheonan, Republic of Korea; ^4^Department of Otorhinolaryngology-Head and Neck Surgery, Chuncheon Sacred Heart Hospital, Chuncheon, Republic of Korea; ^5^Institute of New Frontier Research, Hallym University College of Medicine, Chuncheon, Republic of Korea

## Abstract

**Methods:**

We isolated T-MSCs from human palatine tonsil and evaluated the ingredients of T-MSCs-CM. The effect of T-MSCs-CM was evaluated in the AR mouse model that was randomly divided into five groups (negative control, positive control, and T-MSCs-CM treated (0.1 mg, 1 mg, and 10 mg)). To investigate the therapeutic effect, we analyzed rhinitis symptoms, serum immunoglobulin (Ig), inflammatory cells, and cytokine expression. We also assessed T cell receptor signal, including MAP kinase (ERK/JNK), p65, and NFAT1.

**Results:**

We identified the increment of TGF-*β*1, PGE2, and HGF in the T-MSCs-CM. In an animal study, the T-MSCs-CM-treated group showed significantly reduced allergic symptoms and infiltration of eosinophils and neutrophils in the nasal mucosa, whereas there was no significant difference in total IgE and the OVA-specific IgE level. Additionally, we found that the 10 mg T-MSCs-CM-treated group showed a significantly decreased IL-4 mRNA expression, compared to the (+) Con group. In the analysis of T cell receptor signal, the phosphorylation of MAP kinases, translocation of p65, and activation of NFAT1 were inhibited after T-MSCs-CM.

**Conclusions:**

Our findings suggest that T-MSCs-CM showed a partial immunomodulatory effect on the AR mouse model by the inhibition of T cell activation via MAP kinase, p65, and NFAT1.

## 1. Introduction

Allergic rhinitis (AR) is a common chronic nasal disease presented by the symptoms of sneezing, rhinorrhea, itchiness, and nasal congestion [1]. It is characterized by Th2 immune response with an increased influx of eosinophils [1]. To date, various treatment options have been introduced to treat AR patients, including medical treatment, surgery, and antigen-specific immunotherapy [2]. However, the recurrences of symptoms are common problems after drug withdrawal and surgery. Additionally, the antigen-specific immunotherapy has some limitations, such as adverse effect and ineffective outcome [3]. Therefore, new treatment options are required in patients with AR to improve long-term efficacy.

Mesenchymal stem cells (MSCs) are multipotent progenitor cells that are capable of differentiating into various cell types, such as adipocytes, osteoblasts, and chondrocytes [4]. Thus, those could contribute to the maintenance and regeneration of various connective tissues, including the bone, muscle, adipose, and cartilage [5–7]. In addition to the potential for tissue repair, increasing evidences have demonstrated that MSCs also exhibit strong immunomodulation potential via their interaction with T lymphocytes, B lymphocytes, natural killer (NK) cells, and dendritic cells (DC) [8–10]. For this reason, several studies have been demonstrated that MSCs derived from the bone marrow and adipose tissues have the effect of immunosuppressive function on allergic airway inflammation [11–14].

Recently, palatine tonsil tissue was also identified as a source of MSCs and tonsil-derived MSCs (T-MSCs) showed abundant expression of immunomodulatory proteins, compared with MSCs derived from the bone marrow and adipose tissue [15, 16]. Moreover, studies for T-MSCs described an immunomodulatory effect on degenerative or inflammatory diseases [17–20]. Our previous study also demonstrated an immunomodulatory effect of T-MSCs in a mouse model of allergic rhinitis [21]. However, conditioned media released by T-MSCs (T-MSCs-CM) are more feasible for preparation and application than T-MSCs themselves. Therefore, in this study, we investigated the immunomodulatory effect of T-MSCs-CM on the allergic rhinitis mouse model and also evaluated the inhibitory mechanism of T cell activation by T-MSCs-CM.

## 2. Materials and Methods

### 2.1. Isolation of Tonsil-Derived Mesenchymal Stem Cells (T-MSCs) and Preparation of Conditioned Media from T-MSCs

To isolate T-MSCs from human palatine tonsil [21], we harvested tonsil tissue after tonsillectomy and it was cultured in stem cell-enrichment condition, which consisted of RPMI-1640 media (Gibco, Grand Island, NY) with 10% fetal bovine serum (Gibco) and 1% penicillin and streptomycin (Gibco) at a density of 2 × 10^4^ cells/cm^2^. The following day, the cells were switched to serum-free RPMI-1640 media. After 3 days, cell supernatants were collected and centrifuged to remove cell debris. The supernatants were freeze-dried to make the concentration of T-MSCs-CM. T-MSCs between passages 4 and 7 were used for T-MSCs-CM generation. This study was approved by the institutional review board of Dankook University Hospital (2015-005).

### 2.2. Flow Cytometry

T-MSCs were characterized by flow cytometry using surface markers. The following antibodies with recommended dilutions were used: CD90-FITC (clone 5E10; BD Pharmingen, San Jose, CA), CD105-PE (clone 266; BD Pharmingen), CD31 (clone JC70A; Dako, Glostrup, Denmark), CD34 (clone B1–3C5; Millipore, Billerica, MA), CD45-FITC (clone F10–89-4; Abcam, Cambridge, UK), Alexa Fluor 488 anti-mouse IgG (Invitrogen), and Alexa Flour 594 anti-rabbit IgG (Invitrogen).

### 2.3. Experimental Design of Allergic Mouse Model

Twenty female BALB/c mice (4 weeks old, 18–20 g; NarabioTech, Inc., Seoul, Korea) were used and divided into five groups: (-) Con (phosphate-buffered saline- (PBS-) treated group, *n* = 5), (+) Con (AR model group, *n* = 5), and 0.1 mg, 1 mg, and 10 mg T-MSCs-CM groups (T-MSCs-CM-treated groups, each *n* = 5). Briefly, mice in the experimental groups were systemically sensitized with 25 *μ*g of ovalbumin (OVA; Sigma-Aldrich, St. Louis, MO, USA) dissolved in 300 *μ*l of PBS in the presence of 2 mg of aluminum hydroxide gel as an adjuvant by intraperitoneal injection on days 0, 7, and 14. The negative control group was injected with PBS. And then, the mice were immunized with 5 *μ*g OVA by nasal instillation at 21-27 days. PBS was administered to the nasal cavity instead of OVA in the negative control group. In T-MSCs-CM-treated groups, T-MSCs-CM with 20 *μ*l conditioned medium were administered intranasally on days 14 to 18 and 21 to 25 of the experimental period. The mice were sacrificed via cervical dislocation on day 29, and all experiments were repeated three times. The committee on the use and care of animals approved all animal experiments, and we followed strict governmental and international guidelines on animal experimentation (DKU-2014–039).

### 2.4. Evaluation of Symptom Score, Serum Total Immunoglobulin E, and OVA-Specific IgE

After the final OVA challenge on day 28, a blinded observer recorded the frequencies of sneezing and nasal rubbing for a 15-minute period. The mice were sacrificed 24 hours after the last OVA challenge, and tissue samples were collected. After perfusion with 3.7% paraformaldehyde, the heads of the mice from each group were removed en bloc and then fixed in 3.7% paraformaldehyde. After removing the nasal cavity from the head of the remaining mice, the nasal mucosa was meticulously removed by using a small curette. Immediate liquid nitrogen immersion of the nasal mucosa was followed by -70°C storage until further use in reverse transcription-polymerase chain reaction. The serum levels of total and OVA-specific immunoglobulin E (IgE) were measured by a solid phase enzyme-linked immunosorbent assay.

### 2.5. Measurement of Cytokines and Total IgE in Tissue Homogenates

Spleen single-cell suspensions were plated in 24-well tissue culture plates at a final concentration of 3 × 10^6^ cells/ml by using RPMI-1640 media (Gibco, Grand Island, NY). The cells were incubated with OVA in a CO_2_ incubator at 37°C for 72 hours and stored at -70°C until cytokines were measured. Cytokines were assayed in a culture supernatant by using a sandwich enzyme-linked immunosorbent assay kit (R&D Systems, Minneapolis, MN) according to the manufacturer's instructions. After measuring the optical density at 450 nm, the concentrations of interleukin- (IL-) 4, IL-5, IL-6, IL-17, interferon- (IFN-) *γ*, and eotaxin-2 were determined by interpolation from a standard curve, and all data were expressed as nanograms per milliliter.

### 2.6. Quantitative Real-Time PCR

Total RNA was extracted from the nasal mucosa of each mouse group by using the TRIzol reagent kit (Invitrogen, Carlsbad, CA). Equivalent amounts of RNA were reverse transcribed by using the iScript cDNA Synthesis Kit (Bio-Rad Laboratories; Hercules, CA). The messenger RNA (mRNA) expression analysis was performed by using an Applied Biosystems 7500 Real-Time PCR System (Applied Biosystems, Foster City, CA). Corresponding primers and probes for the cytokines and chemokines listed in Table 1 were purchased from Applied Biosystems.

### 2.7. Western Blot Analysis

The BCA kit (Pierceprotein Stint® Inc., BCA Protein Assay Kit, Thermo Scientific USA) was used, a total 50 *μ*g protein of SDS-PAGE was separated through electrophoresis after, and the nitrocellulose membrane (Hybond ECL, GE Healthcare Life sciences, UK) was transferred. The protein is a membrane to transfer 3% skim milk (BD Difco, USA) after each block with diluted primary antibodies phospho-ERK (1 : 1000; Cell Signaling, Danvers, MA, USA), ERK (1 : 2,000, Cell Signaling, Danvers, MA, USA), phospho-JNK (1 : 500; Santa Cruz, CA), JNK (1 : 500; Santa Cruz, CA), p65 (1 : 1,000; Cell Signaling, Danvers, MA, USA), NFAT1 (information), and *β*-actin (1 : 10000; Sigma-Aldrich, USA) antibody overnight. Tris-buffered saline with Tween 20 (+0.1% Tween 20; TBS-T) as a cleanser and secondary antibodies ((p)-ERK, p38, (p)-(p)-p65, HRP-anti-rabbit; p-JNK, HRP-anti-goat; and *β*-actin, JNK, HRP-anti-mouse) triggered the reaction. ECL Western blotting and the detection solution (Amersham detection reagents; GE Healthcare Life Sciences, UK) react with the Gel Doc image analysis system (Bio-Rad, Hercules) which was confirmed using protein bands.

### 2.8. Statistical Analysis

The statistical analyses were performed using GraphPad Prism 4 software. Results are shown as the mean ± SEM. A Mann-Whitney *U* test was used to compare results between groups. A *P* value < 0.05 was considered statistically significant. *P* values < 0.05 were indicated by “^∗^”; *P* values < 0.01 were indicated by “^∗∗^” and < 0.001 by “^∗∗∗^”.

## 3. Results

### 3.1. Characterization of Tonsil-Derived Mesenchymal Stem Cell

**Figure 1:**
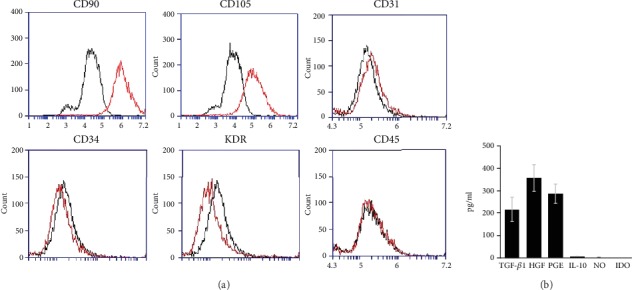
(a) Flow cytometry analysis of surface antigen expression on cultured tonsil-derived mesenchymal stem cells (T-MSCs). Isotype antibody (dark gray color) and experimental antibodies (light gray color) were used. (b) Ingredients of T-MSC conditioned medium were evaluated, including PGE2, TGF-*β*1, HGF, IL-10, NO, and IDO.

To characterize the profile of T-MSCs, we performed flow cytometry analyses (Figure 1(a)). We found that cells were positively labeled with human MSC markers, such as CD90 (Thy-1) and CD105 (endoglin), whereas there was the negative expression for human endothelial cell markers (CD34, CD31, and KDR) and hematopoietic cell marker (CD45). Based on the surface antigen expression, our findings indicated that the expanded cells included a large population of T-MSCs, but these cells did not contaminate with the endothelial cell and hematopoietic cell. Next, we evaluate the ingredients of T-MSCs-CM, which were expected to have immunomodulatory effects. In this study, we measured the concentrations of prostaglandin E2 (PGE2), transforming growth factor beta (TGF-*β*), hepatocyte growth factor (HGF), IL-10, nitric oxide (NO), and indoleamine-pyrrole 2,3-dioxygenase (IDO) (Figure 1(b)). We found that the concentrations of TGF-*β*1, PGE2, and HGF increased more than 200 pg/ml, whereas those of IL-10, NO, and IDO were negligible.

### 3.2. Immunomodulatory Effect of Conditioned Media from Tonsil-Derived Mesenchymal Stem Cell

**Figure 2:**
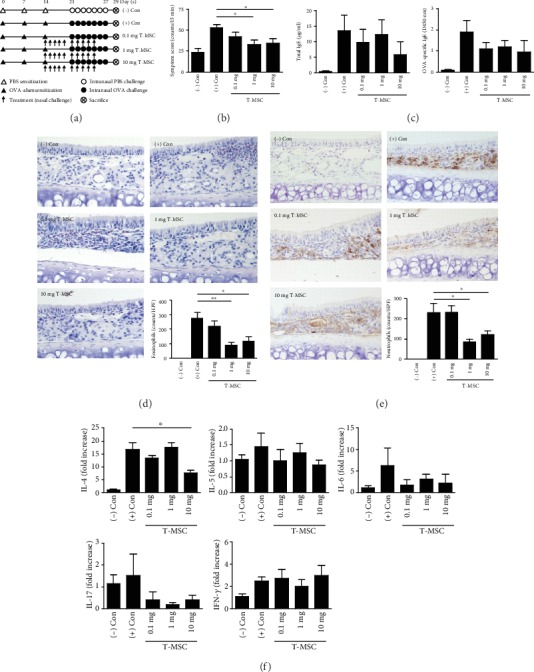
Effect of tonsil-derived mesenchymal stem cells (T-MSCs) on an allergic mouse model. (a) The protocol for generating the murine model of allergic rhinitis. (b) Symptom scores such as sneezing and nasal rubbing were evaluated for 15 minutes after OVA challenge. (c) Total serum and OVA-specific IgE levels were compared among groups. The number of (d) infiltrated, (e) eosinophils, and (f) neutrophils. Cytokine profiles from the nasal mucosa were compared among groups using quantitative real-time polymerase chain reaction.

To investigate the therapeutic potential of T-MSCs-CM in allergic rhinitis, we used a murine model of allergic rhinitis (Figure 2(a)). This murine model showed that the symptom score was observed as 23.6 in the (-) Con, 53.2 in the (+) Con, 42.2 in 0.1 mg T-MSCs-CM-treated, 33.2 in 1 mg T-MSCs-CM-treated, and 35.0 in 10 mg T-MSCs-CM-treated groups. These findings indicated that 1 mg or 10 mg T-MSCs-CM-treated groups showed significantly decreased symptom scores compared with the (+) Con group (Figure 1(b)). However, there was no significant difference in total IgE or OVA-specific IgE between (+) Con and T-MSCs-CM-treated groups (Figure 1(c)). We also observed that the numbers of infiltrated eosinophils and neutrophils were significantly decreased in the 1 mg or 10 mg T-MSCs-CM-treated groups compared to the (+) Con group (Figures 1(d) and 1(e)). Next, to verify the change in the infiltration of eosinophils after treating T-MSCs-CM, we evaluated the mRNA expression of nasal cytokine profiles (Figure 1(f)). The (+) Con group had an increased IL-4, IL-5, IL-6, IL-17, and IFN-*γ* mRNA expression in the nasal mucosa, whereas only the 10 mg T-MSCs-CM-treated group showed a significantly reduced expression of IL-4 mRNA level, compared to the (+) Con group.

### 3.3. Inhibitory Effect of Conditioned Media from Tonsil-Derived Mesenchymal Stem Cells on T Cell Activation

**Figure 3:**
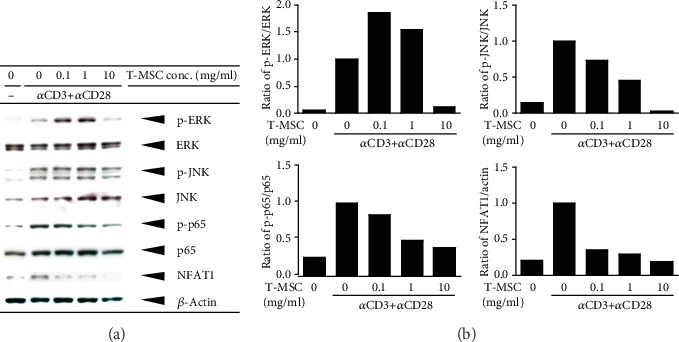
The immunoblotting results of T cell receptor signaling pathways in the human Jurkat T cell. Western blots of cytoplasmic protein with antibodies to ERK, JNK, p65, phospho-ERK, phospho-JNK, phosphor-p65, NFAT1, and *β*-actin. Relative intensities of blot bands were measured with ImageJ software.

To investigate whether T-MSCs-CM could inhibit T cell activation, we assessed T cell receptor signaling, such as MAP kinase (ERK/JNK), p65, and NFAT1 transcription factors in Jurkat T cell stimulated with CD3 and CD28 antibodies (Figure 3). We detected the decreased expression of ERK/JNK phosphorylation, p65 phosphorylation, and NFAT1 activation according to the concentration of T-MSCs-CM on Western blot. Additionally, the phosphorylation of ERK/JNK and P65 was significantly decreased by 10 mg T-MSCs-CM, compared with only anti-CD3/CD28-stimulated Jurkat T cells.

## 4. Discussion

To the best of our knowledge, this is the first study to evaluate the therapeutic effect of T-MSCs-CM in a mouse model for AR. In the present study, we found that T-MSCs-CM showed the increased TGF-*β*1, PGE2, and HGF expressions which are considered immunomodulatory factors. In the animal study, the addition of T-MSCs-CM induced a significantly decreased allergic symptoms, eosinophil infiltration, and Th2 cytokine (IL-4) expression between (+) Con and T-MSCs-CM-treated groups, although there was no effect on the total IgE and OVA-specific IgE level. Additionally, we observed that T cell receptor signaling, such as MAP kinase (ERK/JNK), p65, and NFAT1 was suppressed in the T-MSCs-CM-treated group, compared to the (+) Con group. Therefore, T-MSCs-CM may have a partial immunomodulatory effect on allergic inflammation by the inhibition of T cell activation via MAP kinase (ERK/JNK), p65, and NFAT1 transcription factors.

It has been known that MSCs have the ability to modulate immune responses through surface molecules and soluble mediators, which then assist the cells in avoiding allogeneic reactions for successful implantation [22–24]. The bone marrow and adipose tissues are considered to be major sources of MSCs. Recently, the human palatine tonsil was reported as a source of MSCs, and T-MSCs also have the potential immunomodulatory effect [25]. Contrasted with other sources of MSCs, we could easily acquire human palatine tonsil tissues after tonsillectomies, a commonly performed surgical procedure in children [26]. It means that the human palatine tonsil is an attractive MSC source for clinical applications because tissue collection does not need unnecessary invasive techniques. However, to date, the effect of T-MSCs-CM has not been investigated thoroughly compared to that of T-MSCs themselves. A prior study for chronic colitis model showed that T-MSCs-CM-treated group has an equivalent effect to T-MSCs-treated group regarding the immunomodulatory effect [27]. Consistent with this, the addition of T-MSCs-CM on the AR mouse model showed a similar immunomodulatory effect, compared with the previous study regarding T-MSCs on the AR mouse model. It is a clinically meaningful finding because T-MSCs-CM is easier to prepare and administer to patients.

The family of MAP kinases includes ERK, p38, and JNK. MAP kinase pathways are major pathways induced by T cell receptor stimulation, and they play a major role in the development and function of T cells [28, 29]. The NF-*κ*B/Rel family comprises five members, including p50, p52, p65 (Rel-A), c-Rel, and Rel-B proteins. NF-*κ*B subunits play a specific role in regulating T cell development and effector functions [30, 31]. Among those, the most abundant form of NF-*κ*B activated by pathologic stimuli is the p65:p50 heterodimer [32]. The NFAT family of transcription factors consists of five members (NFAT1–NFAT5), and T cells express three of the four calcium-regulated NFAT proteins, such as NFAT1, NFAT2, and NFAT4. NFAT proteins are activated following T cell receptor ligation, and thus, these proteins are key regulators of T cell differentiation and development [33, 34]. Interestingly, our findings showed that T-MSCs-CM inhibited the phosphorylation of MAP kinase, the migration of p65 to the nucleus, and the decreased activation of the NFAT1 transcription factor. These results suggested that T-MSCs-CM may show the immunomodulatory effect on the allergic inflammatory response via inhibition of T cell activation.

In conclusion, T-MSCs-CM showed a partial immunomodulatory effect in the AR mouse model. Additionally, the ingredient of T-MSCs-CM, such as TGF-*β*1, PGE2, and HGF has an inhibitory effect of T cell activation via the suppression of T cell receptor signal. Therefore, it is expected that administration of T-MSCs-CM, and not the T-MSCs themselves, may exert a therapeutic effect in patients with AR. This application is desirable because it would be easier to perform and would also be accompanied by fewer side effects.

## Figures and Tables

**Table 1:** List of gene-specific TaqMan probes for qRT-PCR.

Gene TaqMan primer	Assay ID	Amplicon length (bp)
IL-4	Mm00445258_g1	63
IL-5	Mm01290072_g1	133
IL-6	Mm00446190_m1	78
IL-17A	Mm00439618_m1	80
IFN-*γ*	Mm99999071_m1	63

## Data Availability

The authors confirm that the data supporting the findings of this study are available within the article.

## References

[1] Bousquet J., Khaltaev N., Cruz A. A. (2008). Allergic Rhinitis and its Impact on Asthma (ARIA) 2008 update (in collaboration with the World Health Organization, GA (2) LEN and AllerGen). *Allergy*.

[2] Bousquet J., Hellings P. W., Agache I. (2019). Allergic Rhinitis and its Impact on Asthma (ARIA) Phase 4 (2018): change management in allergic rhinitis and asthma multimorbidity using mobile technology. *The Journal of Allergy and Clinical Immunology*.

[3] Wise S. K., Lin S. Y., Toskala E. (2018). International consensus statement on allergy and rhinology: allergic rhinitis. *International Forum of Allergy & Rhinology*.

[4] Singer N. G., Caplan A. I. (2011). Mesenchymal stem cells: mechanisms of inflammation. *Annual Review of Pathology*.

[5] Liu T. M., Martina M., Hutmacher D. W., Hui J. H., Lee E. H., Lim B. (2007). Identification of common pathways mediating differentiation of bone marrow- and adipose tissue-derived human mesenchymal stem cells into three mesenchymal lineages. *Stem Cells*.

[6] Uccelli A., Moretta L., Pistoia V. (2008). Mesenchymal stem cells in health and disease. *Nature Reviews Immunology*.

[7] Delorme B., Charbord P. (2007). Culture and characterization of human bone marrow mesenchymal stem cells. *Methods in Molecular Medicine*.

[8] Shi Y., Hu G., Su J. (2010). Mesenchymal stem cells: a new strategy for immunosuppression and tissue repair. *Cell Research*.

[9] Uccelli A., Pistoia V., Moretta L. (2007). Mesenchymal stem cells: a new strategy for immunosuppression?. *Trends in Immunology*.

[10] Ghannam S., Bouffi C., Djouad F., Jorgensen C., Noel D. (2010). Immunosuppression by mesenchymal stem cells: mechanisms and clinical applications. *Stem Cell Research & Therapy*.

[11] Cho K. S., Park H. K., Park H. Y. (2009). IFATS collection: immunomodulatory effects of adipose tissue-derived stem cells in an allergic rhinitis mouse model. *Stem Cells*.

[12] Goodwin M., Sueblinvong V., Eisenhauer P. (2011). Bone marrow-derived mesenchymal stromal cells inhibit Th2-mediated allergic airways inflammation in mice. *Stem Cells*.

[13] Kavanagh H., Mahon B. P. (2011). Allogeneic mesenchymal stem cells prevent allergic airway inflammation by inducing murine regulatory T cells. *Allergy*.

[14] Nemeth K., Keane-Myers A., Brown J. M. (2010). Bone marrow stromal cells use TGF-*β* to suppress allergic responses in a mouse model of ragweed-induced asthma. *Proceedings of the National Academy of Sciences of the United States of America*.

[15] Cho K. A., Kim J. Y., Kim H. S., Ryu K. H., Woo S. Y. (2012). Tonsil-derived mesenchymal progenitor cells acquire a follicular dendritic cell phenotype under cytokine stimulation. *Cytokine*.

[16] Cho K. A., Park M., Kim Y. H., Woo S. Y., Ryu K. H. (2017). RNA sequencing reveals a transcriptomic portrait of human mesenchymal stem cells from bone marrow, adipose tissue, and palatine tonsils. *Scientific Reports*.

[17] Kim J. Y., Park M., Kim Y. H. (2018). Tonsil-derived mesenchymal stem cells (T-MSCs) prevent Th17-mediated autoimmune response via regulation of the programmed death-1/programmed death ligand-1 (PD-1/PD-L1) pathway. *Journal of Tissue Engineering and Regenerative Medicine*.

[18] Kim S. Y., Kim Y. R., Park W. J. (2015). Characterisation of insulin-producing cells differentiated from tonsil derived mesenchymal stem cells. *Differentiation*.

[19] Park H. S., Lee J., Kim J. W. (2018). Preventive effects of tonsil-derived mesenchymal stem cells on osteoradionecrosis in a rat model. *Head & Neck*.

[20] Yu Y., Song E. M., Lee K. E. (2017). Therapeutic potential of tonsil-derived mesenchymal stem cells in dextran sulfate sodium-induced experimental murine colitis. *PloS One*.

[21] Samivel R., Kim E. H., Chung Y. J., Mo J. H. (2015). Immunomodulatory effect of tonsil-derived mesenchymal stem cells in a mouse model of allergic rhinitis. *American Journal of Rhinology & Allergy*.

[22] Aggarwal S., Pittenger M. F. (2005). Human mesenchymal stem cells modulate allogeneic immune cell responses. *Blood*.

[23] Beyth S., Borovsky Z., Mevorach D. (2005). Human mesenchymal stem cells alter antigen-presenting cell maturation and induce T-cell unresponsiveness. *Blood*.

[24] Cui L., Yin S., Liu W., Li N., Zhang W., Cao Y. (2007). Expanded adipose-derived stem cells suppress mixed lymphocyte reaction by secretion of prostaglandin E2. *Tissue Engineering*.

[25] Oh S. Y., Choi Y. M., Kim H. Y. (2019). Application of tonsil-derived mesenchymal stem cells in tissue regeneration: concise review. *Stem Cells*.

[26] Brigger M. T., Brietzke S. E. (2016). Outpatient tonsillectomy in children: a systematic review. *Otolaryngology–Head and Neck Surgery*.

[27] Lee K. E., Jung S. A., Joo Y. H. (2019). The efficacy of conditioned medium released by tonsil-derived mesenchymal stem cells in a chronic murine colitis model. *PloS One*.

[28] Cantrell D. (1996). T cell antigen receptor signal transduction pathways. *Annual Review of Immunology*.

[29] Rincon M., Flavell R. A., Davis R. J. (2001). Signal transduction by MAP kinases in T lymphocytes. *Oncogene*.

[30] Gerondakis S., Strasser A., Metcalf D., Grigoriadis G., Scheerlinck J. Y., Grumont R. J. (1996). Rel-deficient T cells exhibit defects in production of interleukin 3 and granulocyte-macrophage colony-stimulating factor. *Proceedings of the National Academy of Sciences of the United States of America*.

[31] Doi T. S., Takahashi T., Taguchi O., Azuma T., Obata Y. (1997). NF-*κ*B RelA-deficient Lymphocytes: Normal Development of T cells and B cells, impaired production of IgA and IgG1 and reduced proliferative responses. *The Journal of Experimental Medicine*.

[32] Oeckinghaus A., Ghosh S. (2009). The NF-*κ*B family of transcription factors and its regulation. *Cold Spring Harbor Perspectives in Biology*.

[33] Macian F. (2005). NFAT proteins: key regulators of T-cell development and function. *Nature Reviews Immunology*.

[34] Huang G. N., Huso D. L., Bouyain S. (2008). NFAT binding and regulation of T cell activation by the cytoplasmic scaffolding Homer proteins. *Science*.

